# Safety study of permanent pacemaker implantation after TAVI under multiple antithrombotic therapies

**DOI:** 10.1186/s12872-026-05528-y

**Published:** 2026-01-21

**Authors:** Yu Zhou, Keng Cheng, Tao Ge, Changlin Ju

**Affiliations:** 1 Department of Cardiology, The First Affiliated Hospital of Wannan Medical College, Wuhu, Anhui 241000 China; 2 Department of Emergency, The First Affiliated Hospital of Wannan Medical College, Wuhu, Anhui 241000 China

**Keywords:** Transcatheter aortic valve implantation, Permanent pacemaker implantation, Antithrombotic therapy, Pocket hematoma, Bleeding complications

## Abstract

**Background:**

Transcatheter aortic valve implantation (TAVI) is a standard minimally invasive treatment for high-risk patients with severe aortic valve disease. However, the safety of permanent pacemaker implantation (PPI) following TAVI under multiple antithrombotic regimens remains inadequately studied.

**Methods:**

In this single-center retrospective study, 203 patients who underwent TAVI between January 2021 and May 2025 were included. Based on post-TAVI PPI status, patients were categorized into pacemaker (PM, *n* = 35) and non-pacemaker (NPM, *n* = 168) groups. Each group was further stratified by antithrombotic regimen (mono-[MA], dual-[DA], or triple-antithrombotic [TA] therapy). Outcomes, including pocket hematoma, thromboembolic events, and infections, were assessed over a three-month follow-up period.

**Results:**

No significant differences in baseline characteristics were observed between the PM and NPM groups. In the PM group, one death occurred in the TA subgroup. Pocket hematoma and major bleeding each occurred in one patient in the TA subgroup, and lead dislodgement occurred in one patient in the DA subgroup. No significant differences in complication rates were found among antithrombotic subgroups (*P* > 0.05). The use of optimized hemostatic techniques—including electrocautery, hemostatic sponges, and compressive bandaging—was associated with low overall hematoma and infection rates.

**Conclusion:**

PPI following TAVI appears safe under various antithrombotic therapies when accompanied by meticulous hemostatic management. Individualized antithrombotic strategies and standardized perioperative techniques may mitigate bleeding and thrombotic risks in this high-risk population.

## Background

Transcatheter aortic valve implantation (TAVI) has emerged as the preferred minimally invasive treatment for high-risk aortic valve disease patients, particularly those unsuitable for surgical intervention [1, 2]. While initially developed for aortic stenosis, recent advancements in stent technology have expanded TAVI applications to include pure aortic regurgitation cases [3, 4]. However, the procedure’s benefits are tempered by significant post-operative ischemic and hemorrhagic complications, necessitating careful antithrombotic management to maintain therapeutic efficacy.

Current antithrombotic strategies after TAVI remain controversial, with options ranging from monotherapy (MA) to triple therapy (TA) based on individual thrombotic and bleeding risks [5–7]. Dual therapy (DA) may benefit patients with atrial fibrillation or recent coronary stents, while TA is reserved for highest-risk cases, though at the cost of increased bleeding [8, 9]. This complexity is further compounded when permanent pacemaker implantation (PPI) becomes necessary, as antithrombotics significantly elevate risks of pocket hematoma (14.3–26.7%), infection (2–5%), and lead dislodgement (1–3%) [10–12] .

Although our team has previously investigated the incidence, predictors, and long-term prognosis of conduction disturbances and PPI following TAVI [13, 14], the peri-procedural safety of performing PPI under continued multiple antithrombotic therapies remains an unaddressed, high-risk clinical scenario. Therefore, this study specifically investigates PPI safety under different anti thrombotic regimens in post-TAVI patients, addressing a critical evidence gap in current guidelines. By systematically analyzing hematoma rates, thromboembolic events, and infection risks across MA, DA, and TA groups, we aim to establish protocolized management strategies that optimize both thromboprophylaxis and device implantation outcomes.

## Methods

### Study design and patient population

**Fig. 1:**
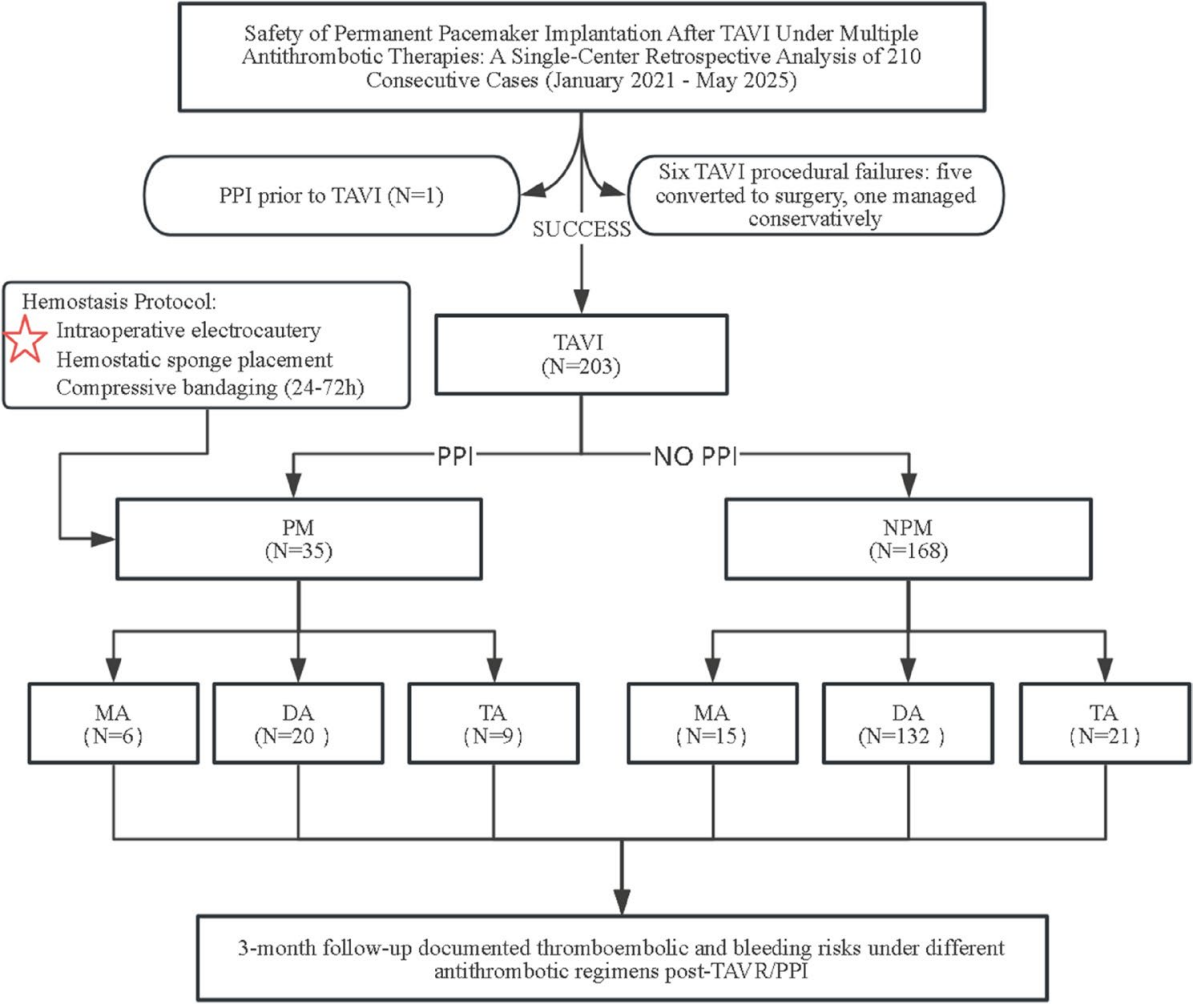
Study Flowchart (A red five-pointed star represents intervention measures)

This single-center retrospective study evaluated the safety of PPI following TAVI in patients with aortic valve disease. From January 2021 to May 2025, patients undergoing transfemoral TAVI at our institution were enrolled. Based on post-TAVI PPI, patients were categorized into a pacemaker group (PM group) and a non-pacemaker group (NPM group). All participants were followed for 3 months to assess the risks of thromboembolism (stroke and TIA), gastrointestinal bleeding, intracranial hemorrhage, pocket hematoma, and pocket infection [15]. Written informed consent was obtained from all patients after detailed explanation of the study purpose and procedures. Exclusion criteria included procedural failure and pre-existing permanent pacemakers. The study complied with the Declaration of Helsinki and was approved by the Institutional Ethics Committee of the Affiliated Hospital of Wannan Medical College (Fig. 1).

### Group stratification

Patients were initially divided into PM and NPM groups based on post-TAVI PPI status. Each group was further stratified according to antithrombotic regimen [16]:

- Monotherapy (MA) group: Receiving either antiplatelet drugs (e.g., aspirin) or anticoagulants (e.g., vitamin K antagonists [VKAs] or direct oral anticoagulants [DOACs]), or single antiplatelet therapy (SAPT).
- Dual therapy (DA) group: Receiving one antiplatelet agent plus one anticoagulant, or dual antiplatelet therapy (DAPT).
- Triple therapy (TA) group: Receiving two antiplatelet agents and one anticoagulant.
- Bleeding and ischemic risks were compared across these subgroups.

### Pre-PPI antithrombotic management

1. *Thromboprophylaxis with Temporary Pacing*: For all patients who required a temporary pacemaker post-TAVI due to conduction disturbances, standardized thromboprophylaxis was instituted to prevent lead-associated thrombosis. This consisted of either therapeutic-dose subcutaneous low-molecular-weight heparin (LMWH, 2000–4000 IU/day) or oral rivaroxaban, continued until the temporary pacemaker was removed or until PPI.
2. For patients with atrial fibrillation on long-term warfarin presenting for TAVI, PPI proceeded without heparin bridging if the INR was < 3.0.
3. Chronic oral antiplatelet therapy (SAPT or DAPT) or DOACs were continued uninterrupted perioperatively whenever possible. A protocol-mandated switch from ticagrelor to clopidogrel was employed due to limited evidence supporting ticagrelor use in the TAVI/PPI perioperative setting.

### Surgical procedures

#### TAVI implantation

All patients underwent comprehensive preprocedural evaluation, including medical history, physical examination, laboratory tests, and imaging, to determine TAVI eligibility. The procedure was performed exclusively using the balloon-expandable VitaFlow Liberty™ system (MicroPort CardioFlow Medtech Corporation). Temporary pacemakers were routinely implanted preoperatively. Procedures were performed under general anesthesia with transesophageal echocardiography guidance to ensure precise valve deployment. Post-implantation hemodynamic assessment determined whether additional interventions were needed. Temporary pacing was maintained for 1–3 days in cases of intraoperative bradycardia, second-degree or higher atrioventricular block, or complete left bundle branch block, with concurrent LMWH subcutaneous injection or continued DOAC/VKA therapy. PPI was performed within one week if conduction abnormalities persisted or worsened.

#### PPI and hemostatic measures

PPI was indicated for persistent high-grade AV block (type II second-degree or higher) or sick sinus syndrome confirmed by the TAVI and electrophysiology teams 3–5 days post-TAVI. To mitigate bleeding risks associated with antithrombotic therapy.

**Fig. 2:**
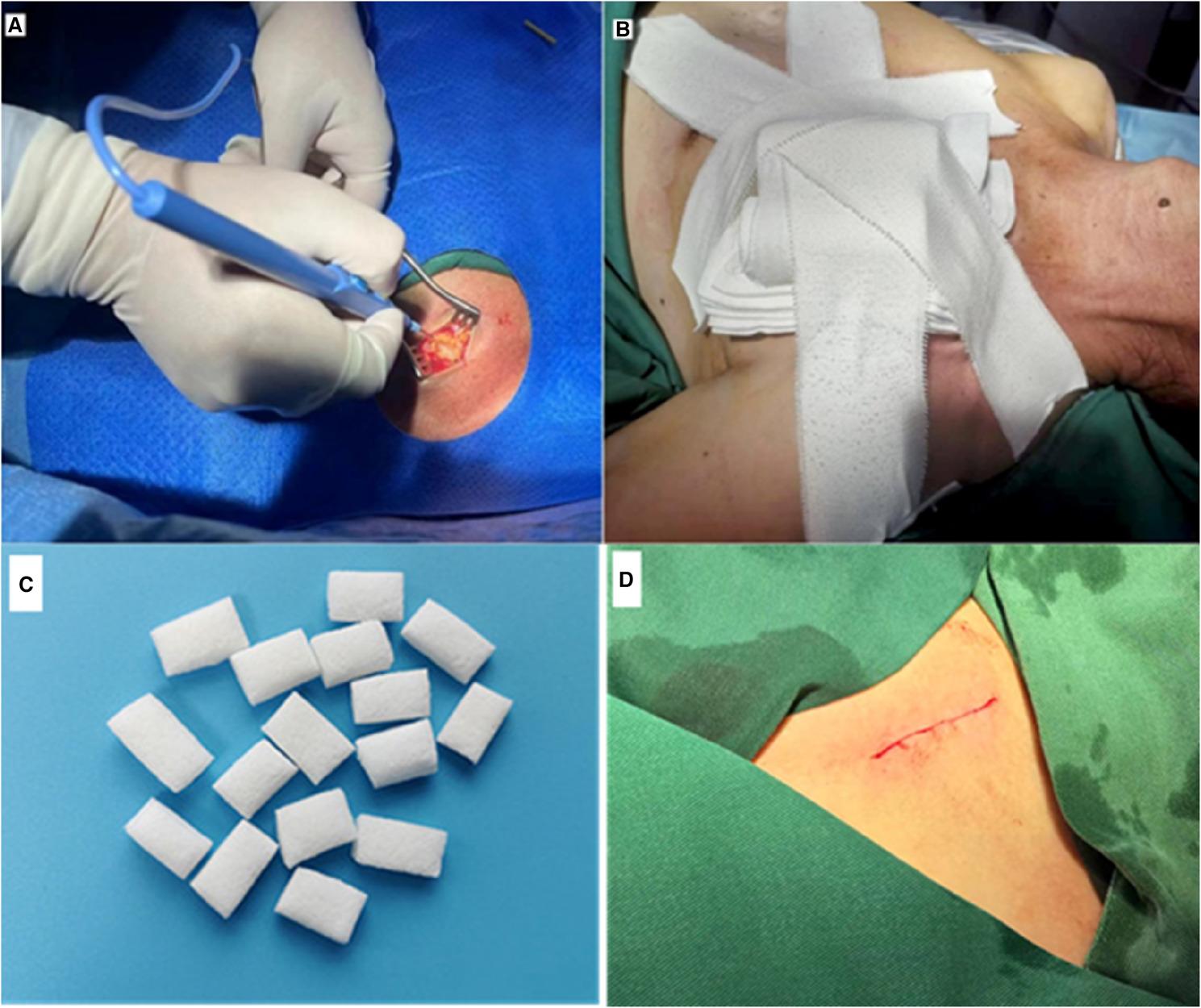
Application of physical hemostatic methods in antithrombotic patients during and after pacemaker implantation (**A**)The use of electric scalpel in pacemaker pocket-making; (**B**) The compression bandage (**C**) Hemostatic sponge (**D**) Wound closure with absorbable sutures

1. Electrosurgical dissection was used for pocket creation to minimize bleeding;
2. Hemostatic sponges were placed in the pocket for high-risk patients (e.g., thin individuals with dissection into muscular layers);
3. Meticulous intraoperative hemostasis (compression or electrocautery) was ensured, followed by compressive bandaging for 24–72 h postoperatively to reduce hematoma risk (Fig. 2).

### Outcome measures

#### Primary endpoint

##### Pocket hematoma

Defined as a clinically significant hematoma at the pacemaker generator site occurring within 3 months post-implantation, characterized by localized swelling, pain, or skin discoloration that required at least one of the following: prolongation of hospitalization, interruption or reversal of antithrombotic therapy, or surgical evacuation.

#### Secondary endpoints

##### Major bleeding

Defined according to the Valve Academic Research Consortium-3 (VARC-3) criteria for major bleeding events [17]. This includes bleeding that is fatal, leads to a drop in hemoglobin of ≥ 3 g/dL, requires transfusion of ≥ 2 units of whole blood or packed red blood cells, results in cardiac tamponade, or requires surgical intervention.

##### Cerebral embolism

Encompasses both ischemic stroke and transient ischemic attack (TIA). Ischemic stroke was defined as a new focal neurological deficit of vascular origin lasting > 24 h and confirmed by neuroimaging (CT or MRI). TIA was defined as a transient episode (< 24 h) of neurological dysfunction with correlating evidence of acute cerebral ischemia on diffusion-weighted MRI.

##### Pocket infection

Defined as infection involving the device pocket, requiring systemic antibiotic therapy and/or surgical intervention (device extraction or debridement), with or without positive microbiological culture.

##### Lead dislodgement

Defined as a displacement of the pacing lead requiring surgical revision due to loss of capture, abnormal sensing thresholds, or radiographic evidence of significant movement from the original implant position.

##### All-cause mortality

Death from any cause within the 3-month follow-up period.

##### Deep Vein Thrombosis (DVT)

Diagnosed by compression ultrasonography showing non-compressibility of a venous segment.

Other Bleeding Events (e.g., Gastrointestinal, Urethral): Documented as clinically overt bleeding requiring medical attention.

### Data collection and statistical analysis

Data on baseline characteristics, procedural details, and postoperative complications were collected from electronic medical records and telephone follow-ups, and verified independently by two researchers. Statistical analyses were performed using IBM SPSS Statistics 26.0. Continuous variables were tested for normality using the Shapiro-Wilk test. Normally distributed data are presented as mean ± standard deviation and compared using t-tests or ANOVA; non-normally distributed data are presented as median (interquartile range) and compared using Mann-Whitney U or Kruskal-Wallis tests. Categorical variables are presented as number (percentage) and compared using the chi-square test, with Fisher’s exact test applied when any expected cell count was < 5. A two-sided P-value < 0.05 was considered statistically significant. Given the exploratory nature of this study and the assessment of multiple secondary endpoints across stratified subgroups, no statistical adjustment for multiple comparisons was performed. The reported P-values for secondary endpoints are therefore unadjusted and should be interpreted descriptively. The primary endpoint was pre-specified as pocket hematoma in the PM group. Findings from analyses of secondary endpoints are considered hypothesis-generating and require confirmation in future studies.

## Results

### Baseline characteristics

**Fig. 3:**
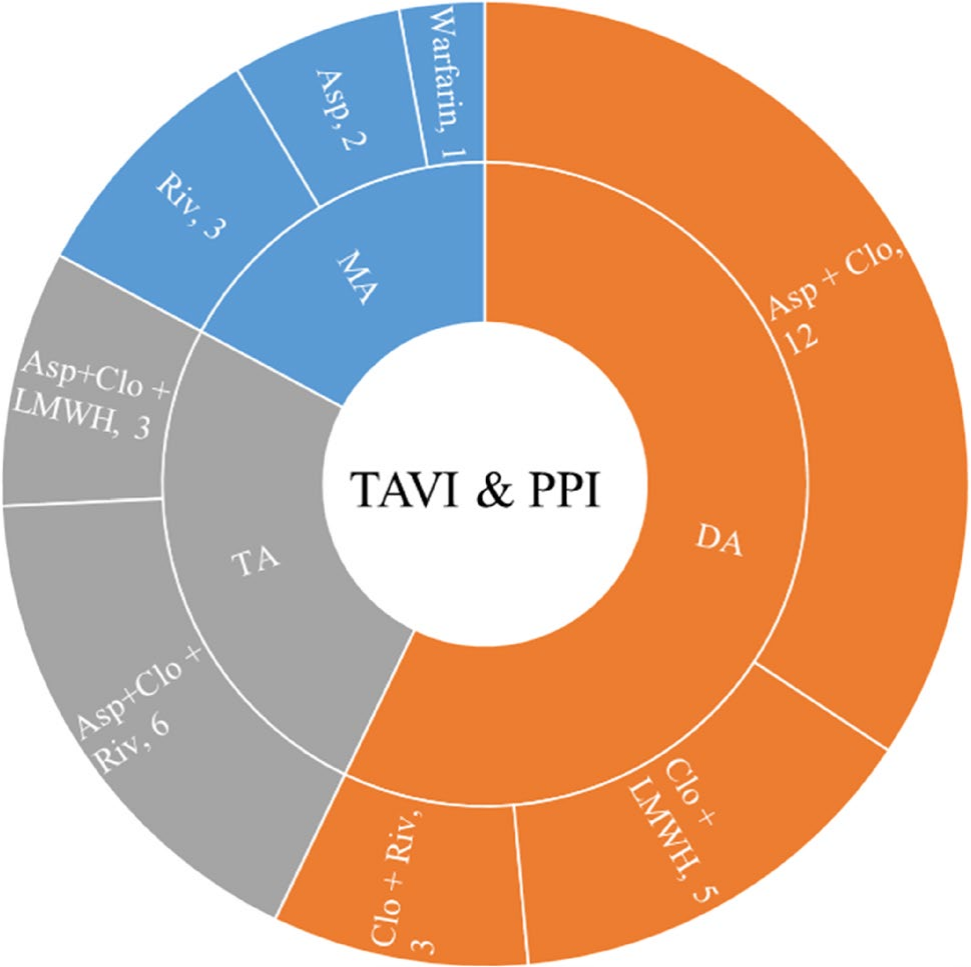
Anticoagulation Therapy Regimens for Permanent Pacemaker Implantation (PPI) in Patients Undergoing Transcatheter Aortic Valve Implantation (TAVI). MA, Mono-Antithrombotic Therapy; DA, Dual Antithrombotic Therapy; TA, Triple Antithrombotic Therapy; Asp, Aspirin; Clo, Clopidogrel; LMWH, Low-Molecular-Weight Heparin

A total of 210 patients with aortic valve disease underwent TAVI. After excluding 6 cases of procedural failure and 1 patient with a pre-existing permanent pacemaker, 203 patients were included in the final analysis. Based on post-TAVI PPI, patients were divided into a PM group (*n* = 35) and a NPM group (*n* = 168). No significant differences were observed between the two groups in demographic characteristics such as age, sex, or BMI (*P* > 0.05). Biochemical parameters, including creatinine, uric acid, blood glucose, and lipid profiles, also showed no significant intergroup differences (*P* > 0.05). Clinical features such as hypertension, coronary artery disease, atrial fibrillation, history of cerebral infarction, and mitral regurgitation were similarly comparable between groups (*P* > 0.05), However, the prosthesis size (Φmm) was significantly larger in the PM group compared to the NPM group (28.56 ± 2.20 mm vs. 26.45 ± 2.72 mm, *P* = 0.001), indicating a potential association between larger valve size and the need for PPI. Apart from this parameter, the baseline characteristics were well-balanced between the two groups (Table 1). The distribution of antithrombotic strategies in the PM group is illustrated in Fig. 3.

**Table 1:** Baseline characteristics of study participants

Parameter	PM Group (*N* = 35)	NPM Group (*N* = 168)	*P*-value
Age (years)	72.60 ± 6.87	72.94 ± 7.82	0.843
Male (n, %)	19 (54.29%)	93 (55.36%)	0.879
Body Mass Index (kg/m²)	22.02 ± 2.93	23.05 ± 2.67	0.862
Creatinine (µmol/L)	78.20 (58.80, 103.90)	74.00 (61.50, 103.70)	0.941
Uric Acid (mmol/L)	381.98 ± 146.22	387.51 ± 142.15	0.853
Blood Glucose (mmol/L)	5.09 ± 0.82	5.12 ± 1.10	0.893
Total Cholesterol (mmol/L)	3.93 ± 0.95	3.88 ± 1.23	0.842
Triglycerides (mmol/L)	1.15 ± 0.65	1.15 ± 0.83	0.978
HDL-C (mmol/L)	1.38 ± 0.29	2.16 ± 0.92	0.661
LDL-C (mmol/L)	2.16 ± 0.72	2.37 ± 0.41	0.541
Lipoprotein(a)	98.70 (68.90, 233.60)	169.10 (81.70, 362.00)	0.252
Prothrombin Time (s)	12.74 ± 1.27	12.32 ± 1.05	0.683
International Normalized Ratio (INR)	1.10 ± 0.11	1.12 ± 0.17	0.842
APTT (s)	35.32 ± 22.13	35.44 ± 23.07	0.856
Platelet Count (×10⁹/L)	134.20 ± 47.03	168.40 ± 50.06	0.271
Hypertension (n, %)	17 (48.57%)	73 (43.45%)	0.579
Coronary Artery Disease (n, %)	8 (22.86%)	30 (14.29%)	0.411
Atrial Fibrillation (n, %)	12 (34.29%)	36 (23.21%)	0.169
History of Cerebral Infarction (n, %)	3 (8.57%)	8 (4.76%)	0.062
Mitral Regurgitation (n, %)	10 (28.57%)	35 (20.83%)	0.179
Prosthesis size (Φmm)	28.56 ± 2.20	26.45 ± 2.72	0.001

Data presented as mean ± standard deviation, median (interquartile range), or number (percentage). *APTT* Activated Partial Thromboplastin Time, *INR* International Normalized Ratio, *HDL-C* High-Density Lipoprotein Cholesterol, *LDL-C* Low-Density Lipoprotein Cholesterol

### Primary endpoint: incidence of pacemaker pocket hematoma

Regarding the pre-specified primary endpoint of pocket hematoma within 3 months after PPI, one event occurred among the 35 patients in the PM group, yielding an overall incidence of 2.9% (1/35). This single case occurred in theTA therapy subgroup (1/9, 11.1%). No pocket hematomas were observed in the MA or DA therapy subgroups. Formal statistical comparison across antithrombotic subgroups was not performed due to the low event rate; however, the observed incidence in this cohort was low (Table 2).

**Table 2:** Baseline and procedural characteristics of patients in the pacemaker after TAVI

Characteristic	Mono-Therapy (MA) (*N* = 6)	Dual Therapy (DA) (*N* = 20)	Triple Therapy (TA) (*N* = 9)	*P*-Value
Age, years	71.83 ± 7.12	72.52 ± 6.48	73.78 ± 7.89	0.891
Male sex, n (%)	3 (50.00)	11 (55.00)	5 (55.56)	0.976
Body Mass Index, kg/m²	22.52 ± 3.02	21.89 ± 2.81	21.78 ± 3.21	0.912
Creatinine, µmol/L	76.52 (60.23, 98.25)	78.75 (58.01, 105.47)	80.08 (59.53, 110.19)	0.943
Platelet Count, ×10⁹/L	140.15 ± 45.12	132.75 ± 47.53	130.12 ± 50.32	0.874
LV Ejection Fraction, %	55.82 ± 5.24	54.31 ± 6.03	53.89 ± 7.12	0.812
Clinical Comorbidities				
Hypertension, n (%)	3 (50.00)	9 (45.00)	5 (55.56)	0.877
Coronary Artery Disease, n (%)	1 (16.67)	4 (20.00)	3 (33.33)	0.621
Atrial Fibrillation, n (%)	2 (33.33)	7 (35.00)	3 (33.33)	0.998
History of Stroke/TIA, n (%)	0 (0.00)	1 (5.00)	2 (22.22)	0.189^Δ^
Chronic Kidney Disease*, n (%)	1 (16.67)	3 (15.00)	2 (22.22)	0.898
Pacing Mode				
VVI (Single-chamber)	1 (16.67)	8 (40.00)	1 (11.11)	0.219
DDD (Dual-chamber)	5 (83.33)	10 (50.00)	8 (88.89)	0.076
CRT (Cardiac Resynchronization Therapy)	0 (0.00)	2 (10.00)	0 (0.00)	0.451
Complications				
Pocket hematoma	0 (0.00)	0 (0.00)	1 (11.11)	0.226^Δ^
Pocket infection	0 (0.00)	0 (0.00)	0 (0.00)	-
Lead dislodgement	0 (0.00)	1 (5.00)	0 (0.00)	0.680^Δ^
Cardiac tamponade	0 (0.00)	0 (0.00)	0 (0.00)	-
Major bleeding	0 (0.00)	0 (0.00)	1 (11.11)	0.226^Δ^

Data presented as mean ± standard deviation, median (interquartile range), or number (percentage). ^Δ,,^, Fisher’s exact test

### Baseline characteristics and device details in the PM group by antithrombotic regimen

To assess potential confounding across antithrombotic subgroups within the PM group, we compared key baseline and procedural characteristics, which are summarized in Table 2. No statistically significant differences were detected among the MA, DA, and TA subgroups in demographics, clinical comorbidities, laboratory values, left ventricular function, or the distribution of pacing modes (VVI, DDD, CRT) (all *P* > 0.05). This relative balance suggests that the observed outcome differences are less likely to be solely attributable to major baseline disparities.

### Secondary and exploratory clinical outcomes

Secondary and exploratory outcomes, including bleeding, thromboembolic events, mortality, and other PPI-related complications (Tables 2 and 3) are summarized below. Between-group comparisons (PM vs. NPM) and exploratory subgroup analyses by antithrombotic regimen are presented. All reported P-values for these endpoints are unadjusted for multiple comparisons and should be interpreted as descriptive and hypothesis-generating.

**Table 3:** Bleeding and embolic complications in patients with and without pacemaker implantation after TAVI

Complications	PM Group				NPM Group				*P*-value
	MA (*N* = 6)	DA (*N* = 20)	TA (*N* = 9)	P-value	MA (*N* = 15)	DA (*N* = 132)	TA (*N* = 21)	P-value	
Mortality (n)	0(0)	0(0)	1(11.11)	0.226	0(0)	3(2.27)	5(23.81)	0.000^**^	0.618
GI Bleeding (n)	0(0)	0(0)	1(11.11)	0.226	0(0)	2(1.52)	2(9.52)	0.067	0.869
Intracranial Hemorrhage (n)	0(0)	0(0)	0(0)	0(0)	0(0)	1(0.76)	1(4.76)	0.263	0.517
Urethral Bleeding (n)	0(0)	1(5.00)	0(0)	0.68	0(0)	0(0)	1(4.76)	0.030^*^	0.218
Cerebral Embolism (n, new)	0(0)	0(0)	1(11.11)	0.226	1(0.67)	1(0.76)	0(0)	0.117	0.457
DVT (n)	1(16.67)	1(5.00)	0(0)	0.219	3(20.00)	1(0.76)	0(0)	0.000^**^	0.289
TIA (n)	1(16.67)	2(10.00)	0(0)	0.497	2(13.33)	5(3.79)	0(0)	0.128	0.273

*PM* Pacemaker, *Non-PM* Non-Pacemaker, *MA* Mono-Antithrombotic Therapy, *DA* Dual Antithrombotic Therapy, *TA* Triple Antithrombotic Therapy, *GI* Gastrointestinal, *DVT* Deep Vein Thrombosis, *TIA* Transient Ischemic Attack; Data presented as n (%), Fisher’s exact test; *P < 0.05, **P < 0.01; interpretation is limited by low event counts

### Comparisons between PM and NPM groups

A comparative overview of key clinical events within the 3-month follow-up period is provided in Table 3. No statistically significant differences were detected between the PM and NPM groups in the rates of all-cause mortality, major bleeding (gastrointestinal or intracranial), cerebral embolism, or TIA. The incidence of DVT was numerically higher in the NPM group, but this difference did not reach statistical significance in this cohort.

### Exploratory subgroup analyses by antithrombotic regimen

Outcomes in the PM Group (*n* = 35), Complications specifically related to the pacemaker device are detailed in Table 2. Aside from the primary endpoint (pocket hematoma), one case of major bleeding and one case of lead dislodgement were recorded in the TA and DA subgroups, respectively. No pocket infections or cardiac tamponade occurred. The choice of pacing mode (VVI, DDD, or CRT) did not differ significantly among the MA, DA, and TA subgroups.

Clinical events such as mortality and thromboembolism in the PM group, stratified by antithrombotic therapy, are included in the combined Table 2. One death and one cerebral embolism occurred, both in the TA subgroup. No statistically significant differences in these event rates were detected across antithrombotic subgroups Table 3, although these analyses are severely underpowered.

Outcomes in the NPM Group (*n* = 168), The distribution of clinical events in the NPM group varied among antithrombotic subgroups (Table 3). A nominally significant difference in all-cause mortality was observed (*P* = 0.000), with events occurring in the DA (3/132, 2.3%) and TA (5/21, 23.8%) subgroups. Similarly, a trend toward a difference in the rate of DVT was noted (*P* = 0.000), with a higher incidence in the MA subgroup (3/15, 20.0%). These unadjusted comparisons, driven by a small number of events, are exploratory and require cautious interpretation. No significant differences were found for other bleeding or ischemic endpoints among subgroups in this cohort.

## Discussion

As the preferred treatment for high-risk aortic valve disease patients, post-TAVI management—particularly optimization of antithrombotic strategies—remains a central clinical concern. With expanding TAVI indications and increasing patient complexity, the rate of PPI has risen significantly. The safety of PPI under multiple antithrombotic regimens requires urgent validation. This retrospective analysis of 203 TAVI patients systematically evaluated the impact of different antithrombotic regimens on PPI-related complications, providing important clinical evidence combined with intraoperative hemostatic techniques. Current post-TAVI antithrombotic strategies largely follow coronary intervention guidelines, lacking specific studies focused on PPI patients. In antiplatelet therapy after TAVI, balancing bleeding and thrombotic risks is essential: DAPT may be suitable for younger patients with high thrombotic risk and low bleeding risk [18], while SAPT is preferred for elderly patients at high bleeding risk. Studies indicate that aspirin alone reduces bleeding risk without compromising prevention of ischemic events compared to DAPT[19–22]. For TAVI patients with atrial fibrillation requiring anticoagulation, two key issues exist [23]: First, the optimal anticoagulant strategy between VKAs and DOACs remains debated. Although TAVI patients were initially excluded from DOAC trials, recent studies show no significant differences in thromboembolism, bleeding, or mortality between DOACs and VKAs [24, 25]. Some studies even suggest lower bleeding and mortality risks with DOACs, making them increasingly considered safer alternatives [26, 27]. Second, heparin bridging, commonly used perioperatively, is associated with higher hematoma and bleeding risks and is not recommended by current guidelines. However, in this study, although LMWH or DOACs were routinely used post-TAVI to prevent DVT, discontinuation of LMWH 12 h before PPI did not increase bleeding or hematoma incidence, likely due to electrocautery use during surgery and compressive dressing application postoperatively.

Combined antiplatelet and anticoagulant therapy is common post-TAVI, especially in patients with recent PCI and atrial fibrillation. Studies in TAVI patients found higher bleeding risks with combination anticoagulant and SAPT therapy compared to anticoagulation alone [28, 29]. The ENVISAGE-TAVI trial showed that among TAVI patients with recent PCI (≤ 90 days) and atrial fibrillation, 37.1% received DAPT; compared to atrial fibrillation patients without recent PCI receiving only SAPT, no significant differences in adverse clinical events were observed, but major organ bleeding risk was significantly higher in the recent PCI group [8]. Compared to OAC plus SAPT, OAC plus DAPT was associated with higher bleeding risk [30, 31]. However, some studies indicate that OAC plus SAPT improves post-TAVI survival [32]. Similarly, in this study, combined DAPT and anticoagulant therapy did not significantly increase pocket hematoma risk. Our findings partially align with the ENVISAGE-TAVI trial: TA subgroup showed increased bleeding events, while the MA subgroup had elevated thrombotic risk. This suggests that antithrombotic regimens should be dynamically adjusted based on individualized risk assessment (e.g., thrombotic burden, bleeding tendency). For example, MA (e.g., aspirin alone) may be optimal for high bleeding risk patients, while DA or TA regimens remain necessary for those with atrial fibrillation or recent PCI at high thrombotic risk.

Antithrombotic therapy increases perioperative risks for PPI in TAVI patients. Beyond optimizing preoperative antithrombotic management, intraoperative hemostatic measures are critical for preventing pocket hematoma. Traditional methods like mechanical compression and suturing often prove inadequate in anticoagulated patients. With advances in minimally invasive techniques, electrocautery has become the preferred tool for intraoperative hemostasis. By using high-frequency current to simultaneously cut and coagulate tissue, electrocautery effectively reduces intraoperative bleeding. Particularly when handling vessels around the pocket, it precisely coagulates small vessels, avoiding bleeding points missed by traditional compression, significantly reducing intraoperative bleeding risk. In this study, all PPI patients underwent pocket creation using electrocautery. Results showed no pocket hematomas even under multiple antithrombotic regimens, strongly demonstrating electrocautery’s efficacy in reducing bleeding risk and ensuring pocket stability. Additionally, electrocautery reduced operation time and patient discomfort, further enhancing safety and efficiency [33]. Postoperative hemostasis remains crucial under antithrombotic therapy. Compression bandaging, a simple and effective physical method, applies sustained pressure to reduce blood accumulation in the pocket, lowering hematoma risk [34]. This study effectively addressed limitations of traditional methods through intraoperative electrocautery and postoperative compression. Electrocautery reduced intraoperative oozing by coagulating vessels with high-frequency current, while compression bandaging suppressed blood accumulation through sustained pressure. These measures maintained a low hematoma rate (11.11%) even in high-intensity antithrombotic therapy (e.g., TA subgroup), consistent with Alkhalil et al.‘s view that optimized hemostatic techniques can balance antithrombotic risks [35]. Additionally, compression bandaging reduces dead space within the pocket, promotes tissue healing, lowers infection risk, facilitates early ambulation, and prevents DVT, thereby improving patient experience and quality of life.

The observed pocket hematoma rates in this study (2.9% overall; 11.1% in the triple-therapy subgroup) are numerically lower than those typically reported in classic CIED studies under anticoagulation (5–15%) or multiple antithrombotic therapy (14–27%) [10, 30]. While cross-trial comparisons require caution, these outcomes suggest that the systematic hemostatic measures employed-electrocautery, hemostatic sponges, and compression bandaging-may help attenuate the bleeding risk in this high-risk population. This experience highlights the potential clinical value of standardizing technical protocols to improve safety when implanting devices in patients requiring intensive antithrombotic therapy.

### Limitations

This study has several limitations. First, its single-center, retrospective design may introduce selection bias and unmeasured confounding factors. Second, the sample size is relatively small, particularly in the pacemaker subgroup (*n* = 35), which limits the statistical power to detect clinically meaningful differences between antithrombotic regimens. Third, the short-term (3-month) follow-up period precludes assessment of long-term device-related outcomes. Finally, it is worth noting that our study was conducted during the COVID-19 pandemic (2021–2023). While pandemic-related constraints could have influenced patient selection and procedural timing, recent evidence suggests that streamlined perioperative pathways allowed TAVI to be performed without compromising early safety outcomes compared to the pre-pandemic period [36]. Future large-scale, prospective, multi-center studies with longer follow-up are warranted to validate these findings.

## Conclusion

In this limited cohort, PPI after TAVI appeared to be feasible under multiple antithrombotic regimens, with no significant increase in bleeding or thromboembolic events observed. Optimized physical hemostatic measures—including intraoperative electrocautery, hemostatic sponge placement, and postoperative compression bandaging—were associated with low rates of pocket hematoma and infection. These preliminary findings suggest that through standardized hemostatic techniques and individualized antithrombotic strategies, PPI may be considered in high-risk TAVI patients requiring multiple antithrombotic therapies. However, the small sample size and low event rates preclude definitive conclusions on safety equivalence; therefore, these results warrant validation in larger, adequately powered studies.

## Data Availability

The data that support the findings of this study are available from the corresponding author, upon reasonable request.
